# An Improved Cr-EA-IRMS Method for the Effective Determination of the Hydrogen Isotopes in Coal Samples

**DOI:** 10.3389/fchem.2022.840473

**Published:** 2022-04-07

**Authors:** Hongwei Li, Lianjun Feng

**Affiliations:** ^1^ Key Laboratory of Mineral Resources, Institute of Geology and Geophysics, Chinese Academy of Sciences, Beijing, China; ^2^ Innovation Academy for Earth Science, Chinese Academy of Sciences, Beijing, China

**Keywords:** Cr-EA-IRMS, isotopes analysis, hydrogen isotope, mass spectrometry, coal samples

## Abstract

Hydrogen isotope analysis of coal is an important tool in the geochemical analysis of coal. The traditional method of hydrogen isotope analysis of coal requires the oxidation of organic matter bound hydrogen in coal to water by an oxidizing agent and then its reduction to hydrogen by a reducing agent. This method is time-consuming and laborious, and makes it difficult to cope with the rapid detection of large numbers of samples. The recent development of continuous flow IRMS systems (CF-IRMS) has solved the problem of inefficient analysis, but does not guarantee the quantitative conversion of organic bound H to H_2_, resulting in inaccurate measured hydrogen isotope values. In this study, for the hydrogen isotope analysis of coal, an alternative continuous flow system (Cr-EA-IRMS) was used to achieve high precision hydrogen isotope measurements of coal samples by filling a quartz reaction tube with Cr. The results obtained by this method (−121.3 ± 1.1‰) for the reference material (GBW11104) are consistent with those obtained by the conventional method (−121.4 ± 0.6‰). Using this method, hydrogen isotope measurements for a variety of imported coals revealed significant differences in the hydrogen isotopes of coals from different coal producing regions including Russia, South East Asia, and Australia. Therefore, the use of hydrogen isotope testing analysis of coal could be a potential means of tracing the origin of coal.

## 1 Introduction

Organic components are the most important constituents of coal. Therefore, the main elemental composition of coal is also composed of the elements carbon, oxygen, nitrogen, hydrogen, and sulfur. Stable isotope studies of each element have also received extensive attention. The hydrogen isotopes of coal are directly related to the environment in which plants grow, such as the hydrogen isotopic composition of groundwater, temperature, and moisture content. The late diagenesis of geological burial can further cause changes in the hydrogen isotopes of coal (Sun and Wang, 1996). There are differences in isotopic characteristics of different types of coal in different producing areas. Studying these differences can help us trace the sites of the coals (Redding et al., 1980).

Isotope ratio mass spectrometry (IRMS) has been widely used in many disciplines which require the ability to measure variations accurately and precisely in isotopic ratios of light elements such as ^13^C/^12^C, ^18^O/^16^O, ^2^H/^1^H, and ^34^S/^32^S in recent years (Muccio and Jackson, 2009). This technique makes it possible to measure the difference in isotope ratio between given samples (Niu et al., 2017).

The dual inlet method is used as a conventional technique for determining the hydrogen isotope ratio of organic compounds in coal. Prior to testing, the hydrogen bound to the organic matter in the coal needs to be converted to water first. Conventionally, an oxidizing agent such as CuO is added to the sample and then the hydrogen in the organic matter is converted to water by heating under vacuum conditions. This is followed by purification to collect the water, which is then transferred to a metal reducer furnace (e.g., chromium powder or zinc pellets, uranium metal) to produce hydrogen gas (Schiegl and Vogel, 1970; Vennemann and O’Neil, 1993; Li et al., 2015). Finally, the hydrogen produced by the reaction is collected for hydrogen isotope determination by dual inlet IRMS. This off-line hydrogen isotope preparation system coupled with dual inlet IRMS analysis gives highly accurate hydrogen isotope analysis results. However, the disadvantages of this off-line method are also obvious. One is that it is time-consuming and labor-intensive; another is that the precision and accuracy are poor for samples with small sample sizes (Li et al., 2015). Later, Gehre M. improved sample preparation device for quantitative hydrogen isotope analysis using chromium metal. Advantages of the new technique included 1) using only microliter quantities of original liquid samples of different chemical composition and origin as well as measuring hydrogen-containing gases like methane, 2) measuring of δ^2^H values within an 1‰ reproducibility and without memory effect, and 3) the fact that use of an autosampler can make this method more effective (Gehre et al., 1996). However, this method was still a dual-inlet method, mainly focusing on water and hydrogen-containing gases.

In recent years, continuous flow isotope ratio mass spectrometry (CF-IRMS) has been widely used for the analysis of stable isotopes. For example, in high-temperature conversion (HTC) systems, hydrogen-containing substances such as water and organic matter are converted to hydrogen gas in a carbon reactor. This hydrogen is then carried by a helium carrier gas into the mass spectrometer for hydrogen isotope measurements. However, this system has the obvious disadvantage that the conversion of hydrogen-containing substances to hydrogen is not completely quantitative, resulting in measurement errors (Gehre et al., 2015).

Recently, the Cr-EA-IRMS method has been widely applied to the hydrogen isotope analysis, effectively avoiding this persistent problem of hydrogen isotope fractionation caused by the incomplete quantitative conversion of HTC systems. Variations of this method have been previously applied to organic compounds (Gehre et al., 2015; Nair et al., 2015; Renpenning et al., 2015) halogen-and sulfur-bearing organic compounds (Gehre et al., 2017) and hydrous minerals and waters (Qi et al., 2017). Hydrogen isotopes analysis of apatite, inclusions and halite aqueous inclusions have also been reported with Cr-HTC-IRMS method (Greenwood, 2018; FourelFrançoisLécuyer and Seris, 2019; Li and Feng, 2019). Elementar offers a commercial chromium reduction system (HDChrome), which provides a more optimized option for hydrogen isotope analysis of hydrogen-containing substances. While such systems have analyzed water and large amounts of hydrogen-containing organic matter, the application of such systems to the hydrogen isotope analysis of coal has not been reported. In this study, we utilized the Thermo Fisher Scientific EA system, which does not offer a system similar to Elementar’s chromium reduction system. It is on the basis of the Thermo Fisher Scientific EA system that we have built the Cr-EA-IRMS system and applied it to the hydrogen isotope analysis of coal.

## 2 Experimental Section

### 2.1 Material

Coal samples were loaded into 5 × 3.5 mm tin capsules (Thermo Fisher Scientific, Bremen, Germany). High-purity helium, and hydrogen gas (purity >99.999%, all from Air Products, Beijing, China) were used as carrier gas, and working standard gas, respectively. Chromium powder (Merck, Darmstadt, Germany, <0.315 mm), chromium chips (Sigma-Aldrich, St Louis, MO, United States, thickness: 2 mm), quartz chips, and quartz wool (Thermo Fisher Scientific, Bremen, Germany) were filled into an empty quartz glass tube to form an elemental analyzer (EA) system.

### 2.2 Coal Samples

GBW11104 (Anthracite, content:S-1.13%, C-79.13%, H-2.23%, N-1.13%, ash-13.80%, volatile-7.02%) purchased from the China National Standards Center was chosen as a coal reference material (RM) to optimize hydrogen isotope analysis. In addition, 39 coal samples from three coal-producing regions including Southeast Asia (Indonesia *n* = 26, Malaysia *n* = 1, and Philippines *n* = 1), Australia (*n* = 5), and Russia (*n* = 6) were provided by the Shanghai Customs District, and the isotopic characteristics from the different coal producing regions were obtained. Detailed information can be found in Table 2.

### 2.3 Hydrogen Isotope Analysis

All the hydrogen isotope results are reported in units permil (‰) with respect to V-SMOW (Vienna Standard Mean Ocean Water). The hydrogen isotope ratio was represented by the δ^2^H value (Li and Feng, 2019):

δ^2^H = [(^2^H/^1^H)_sample_/(^2^H/^1^H)_vsmow_−1] × 1,000 (CoplenT, 2011).

USGS70 (eicosanoic acid methyl ester, δ^2^H = −183.9‰), USGS71 (eicosanoic acid methyl ester, δ^2^H = −4.9‰), and USGS77 (polyethylene powder, δ^2^H = −75.9‰) are used as reference materials for three-point linear normalization in processing raw values for Cr-EA-IRMS method.

#### 2.3.1 Dual-Inlet Method

The traditional off-line combustion of milligram amounts of coal samples with copper oxide (CuO) at 800°C in evacuated and sealed “quartz” ampules overnight offers sufficient time at high temperature to yield quantitatively H_2_O. Cryogenic separation of combustion products in a vacuum line yielded pure H_2_O. The water was then reacted with uranium metal at 800°C to generate H_2_, which was collected on charcoal at −196°C and the isotope ratios of H_2_ were measured.

#### 2.3.2 Cr-EA-IRMS Method

Usually during Cr-EA-IRMS experiments, oxygen needs to be introduced to accelerate the oxidation of reducing substances and to increase the reaction temperature. However, in this experiment no oxygen was injected into the EA. One advantage of not injecting oxygen is that it reduces the rapid consumption of Cr in the EA filled column. At the same time the hydrogen bound to the organic matter in the coal can still reach complete cracking at 1,050°C. This is a significant difference to the process we have used in the past for hydrogen isotope analysis of fluid inclusions (Li and Feng, 2019). In the analysis of hydrogen isotopes in fluid inclusions, the injection of oxygen is conducive to the release of water from the inclusions by thermal decrepitation.

In this method, the filling sequence of the quartz reactor from the bottom to the top of the column is as follows: 20 mm of quartz wool, a ∼120 mm of quartz chips, 10 mm of quartz wool, 50 mm of chromium powder, and 50 mm of chromium chips. An upper 10 mm layer of quartz wool was used to separate the chromium from accumulated residues. The hottest zone of the reactor was occupied by the chromium sector. The reactor temperature was set at 1,050°C, and the GC (gas chromatography) column temperature was maintained at 90°C. A Flash 2000HT elemental analyzer with a MAS 200R autosampler coupled to a Delta V Advantage isotope ratio mass spectrometer *via* a ConFlo IV universal interface (all from Thermo Fisher Scientific) constituted the continuous-flow analysis system for hydrogen analysis. The flow rates of the carrier helium gas and reference gas in the EA were set to 100 ml/min and 180 ml/min, respectively. The solid samples were wrapped in tin capsules and dropped into the furnace using the autosampler.

Protonation reactions in the ion source result in H_3_^+^ ion production (H_2_^+^+H_2_→H_3_^+^+H). The H_3_^+^ portion of the m/z ion beam is determined as H_3_-factor. The H_3_-factor is used to correct the H_3_^+^ contribution to the m/z 3 signal. A low and stable H_3_-factor is needed for a good DH/H_2_ determination. The H_3_-factor determination must be performed before any other procedure. Correction for H_3_^+^ interference was performed mathematically. The correction factor was determined using an automated subroutine (Isodat 3.0 software, Thermo Fisher Scientific). The H_3_^+^ factor was determined twice a day within an interval from 1 to 12 nA in a fully automated fashion and proved to be stable to within 6.8 ± 0.07 ppm nA^−1^ over several weeks.

**FIGURE 1:**
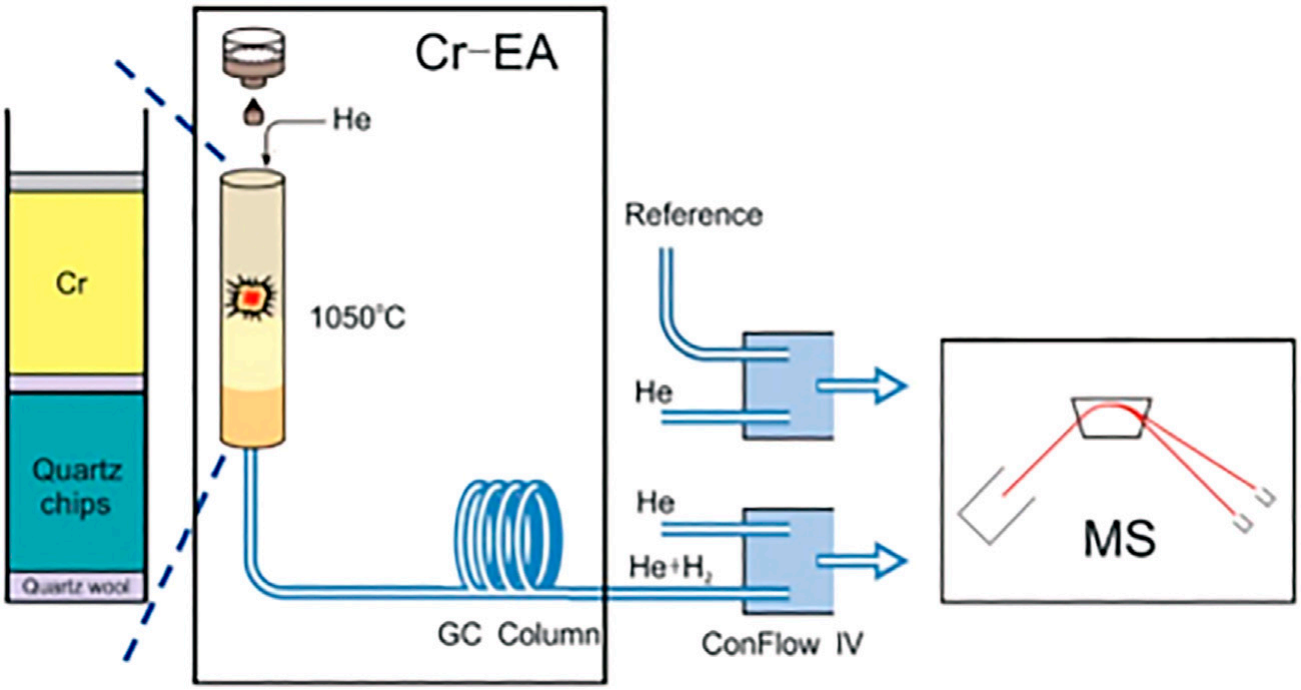
Simplified sketch of the analytical system for hydrogen isotope ratio.

Consequently, this method represents a technical combination of continuous-flow mass spectrometry and Cr-reduction. Furthermore, in order to reduce the influence of background, a U-shaped cold trap filled with molecular sieves (5Å) was installed at the carrier gas outlet to purify helium (Li and Feng, 2019). The schematic diagram of hydrogen isotope determination in coal by improved Cr-EA-IRMS method is shown in Figure 1.

During the analytical procedure, each sample was analyzed three times to ensure the accurate values. A blank tin cup and standard reference were followed after measuring six group of values to verify the stability of the instrument. At the same time, as Cr is consumed during the reaction, new Cr is added to the quartz reaction tube after every 80 samples are analyzed. All raw measurements were referred to pulses of the H_2_ working gas, and data were processed using Isodat 3.0 software (Thermo Fisher Scientific). Multiple-point linear regression based on certified reference materials run during the same sequence was applied to raw δ^2^H values normalization (Li and Feng, 2019).

The method described in this article was used to analyze the hydrogen isotope of the coal components reference material (GBW11104). The results are shown in Table 1. The hydrogen isotope ratio is −121.3 ± 1.1‰ for Cr-EA-IRMS method versus −121.4 ± 0.6‰ for off-line method. In general, no isotopic difference was found between the two methods.

**TABLE 1:** Analyses of δ^2^H of coal reference material (GBW11104).

Reference material	Types of coals	Cr−EA−IRMS	Off−line
		δ^2^H (‰, VSMOW)	δ^2^H (‰, VSMOW)
GBW11104	Anthracite	−122.6	−121.8
	Anthracite	−121.2	−121.0
	Anthracite	−120.6	−121.1
	Anthracite	−121.1	−120.7
	Anthracite	−119.7	−122.2
	Anthracite	−122.5	−121.5
Average		−121.3	−121.4
1σ		1.1	0.6

## 3 Results and Discussion

A Cr-EA-IRMS method was proposed for ^2^H/^1^H analysis of coal samples for the first time. Due to the lack of coal standards with known isotopic composition of the studied components, the hydrogen isotope ratio of GBW11104 (coal reference material of components) is analyzed by Cr-EA-IRMS method and off-line method. The values of the two methods are consistent, which validates of the Cr-EA-IRMS method. The hydrogen isotope ratio of GBW11104 is reported for the first time, which provides a reference material for subsequent analysis.

With respect to wide investigations conducted on δ^2^H values of coal, the hydrogen isotope ratios of coal producing areas in South Asia and Russia have not been reported before. In this study, the δ^2^H values of coal from Indonesia ranged from −150.5 to −106.4‰, with average values of −128.2 ± 13.7‰, and the two Philippine and Malaysian coals were −100.9 and −111.8‰, respectively (Table 2). It is considered that the coal from South Asia has a relatively positive hydrogen isotope ratio. Only a few H isotopes of Russian coal have previously been obtained, and this study shows a narrow distributions of δ^2^H values (−158.5 ± 11.5‰)and δ^2^H values (−211.2 ± 0.9‰) for Eastern Russian coals and Western coals, respectively (Table 2). The Australian coals show similarly negative values (average −163.3 ± 17.0‰) (Table 2). The values of hydrogen isotope of coal samples have a similar trend range as the carbon and nitrogen isotope values, and shows obvious characteristics (Feng et al., 2020).

**TABLE 2:** Analyses of δ^2^H of coals from different regions.

Sample number	Types of coals	Producing region	δ^2^H (‰, VSMOW)
C−1	Bituminous coal	Eastern Russia	−166.6
C−2	Sub bituminous coal	Eastern Russia	−150.4
Average			−158.5
1σ			11.5
C−16	Sub bituminous coal	Western Russia	−212.0
C−44	Bituminous coal	Western Russia	−211.9
C−45	Bituminous coal	Western Russia	−210.7
C−61	Bituminous coal	Western Russia	−210.2
Average			−211.2
1σ			0.9
C−3	Bituminous coal	Australia	−187.2
C−39	Bituminous coal	Australia	−166.8
C−40	Bituminous coal	Australia	−148.4
C−42	Bituminous coal	Australia	−145.4
C−64	Bituminous coal	Australia	−168.9
Average			−163.3
1σ			17.0
C−4	Bituminous coal	Indonesia	−138.9
C−5	Lignite	Indonesia	−138.7
C−6	Lignite	Indonesia	−150.5
C−7	Lignite	Indonesia	−139.5
C−8	Lignite	Indonesia	−150.1
C−9	Lignite	Indonesia	−134.2
C−10	Lignite	Indonesia	−140.1
C−11	Lignite	Indonesia	−133.8
C−12	Lignite	Indonesia	−136.9
C−13	Lignite	Indonesia	−150.1
C−14	Lignite	Indonesia	−143.8
C−15	Lignite	Indonesia	−137.9
C−41	Sub bituminous coal	Indonesia	−109.3
C−43	Sub bituminous coal	Indonesia	−116.2
C−46	Bituminous coal	Indonesia	−119.6
C−47	Lignite	Indonesia	−125.3
C−48	Sub bituminous coal	Indonesia	−119.3
C−49	Sub bituminous coal	Indonesia	−113.0
C−50	Lignite	Indonesia	−106.4
C−51	Sub bituminous coal	Indonesia	−109.1
C−52	Sub bituminous coal	Indonesia	−118.2
C−53	Lignite	Indonesia	−124.1
C−54	Lignite	Indonesia	−124.8
C−55	Sub bituminous coal	Indonesia	−112.0
C−56	Lignite	Indonesia	−121.0
C−57	Sub bituminous coal	Indonesia	−119.9
Average			−128.2
1σ			13.7
C−60	Sub bituminous coal	Malaysia	−111.8
C−62	Sub bituminous coal	Philippines	−100.9

In general, the coal samples from three coal-producing regions show different hydrogen isotopic characteristics. From our analysis results, the hydrogen isotope values of coal from Russia, and that from South Asia are the most and least negative, respectively. The hydrogen isotope values provided by this study can offer a reference for the further in-depth study of coal in different production areas. Meanwhile, this study reports a greater number of H isotope signatures, which can be entered into the database for H isotope research for coal.

## Data Availability

The original contributions presented in the study are included in the article/Supplementary Material, further inquiries can be directed to the corresponding author.
